# Comparability of three intraocular pressure measurement: iCare pro rebound, non-contact and Goldmann applanation tonometry in different IOP group

**DOI:** 10.1186/s12886-019-1236-5

**Published:** 2019-11-14

**Authors:** Min Chen, Lina Zhang, Jia Xu, Xinyi Chen, Yuxiang Gu, Yuping Ren, Kaijun Wang

**Affiliations:** 1 Eye Center, the 2nd Affiliated Hospital, Medical College of Zhejiang University, No.88 Jiefang Road, Hangzhou, 310009 China; 2 Zhejiang Provincial Key Lab of Ophthalmology, Hangzhou, China; 3 Lishui People’s Hospital, Lishui, Zhejiang China; 4 Shaoxing Traditional Chinese Medicine Hospital, Shaoxing, Zhejiang China

**Keywords:** Intraocular pressure, iCare rebound tonometer, Non-contact tonometer, Goldmann applanation tonometer, Central corneal thickness

## Abstract

**Background:**

Measurement of intraocular pressure (IOP) is essential for glaucoma patients. Many factors such as central corneal thickness (CCT) can affect the accuracy of IOP measurement. The purpose of this study was to evaluate the agreement of IOP measured by non-contact tonometer (NCT), iCare pro rebound tonometer (iCare), and Goldmann applanation tonometer (GAT) in different IOP group.

**Methods:**

This was a Hospital-based cross-sectional study. Two hundred subjects were enrolled in this study. All subjects underwent IOP measurement using an NCT–iCare–GAT sequence. Bland-Altman, Pearson correlation and intraclass correlation analysis were performed using SPSS 17.0 software. The influence of CCT on each IOP measurement methods was evaluated by linear regression analysis.

**Results:**

The mean difference (Δ) of NCT–GAT did not differ from (Δ) iCare–GAT in IOP < 10 and 10–21 mmHg group. However, (Δ) NCT–GAT was significantly higher than (Δ) iCare–GAT in IOP 22–30 and > 30 mmHg group (*P* < 0.05). Bland–Altman analysis showed significant agreement between the three devices (*P* < 0.01). IOP measurements of the three methods were significantly correlated with CCT (*P* < 0.01).

**Conclusions:**

ICare pro shows a higher agreement with GAT over a wide range of IOP compared with NCT. The consistency between the three tonometers was similar in a low and normal IOP range. However, NCT shows a greater overestimate of IOP in moderate and higher IOP group. The variability of IOP measurement affected by CCT is NCT > iCare pro > GAT.

## Background

The measurement of intraocular pressure (IOP) is an essential examination in daily ophthalmic procedures, also is a fundamental tool in the management and follow-up of glaucoma patients [1]. Many factors, such as central corneal thickness (CCT), corneal astigmatism and biomechanical properties of the cornea can affect the accuracy of IOP measurement [2–4].

So far, Goldmann applanation tonometry (GAT) is considered as a worldwide accepted gold standard for IOP measurement In clinical practice [5]. Recently, several new methods have been devised, in order to make the IOP measurement faster and more comfortable [6, 7]. Among the alternatives, the iCare Pro rebound tonometer (iCare, Helsinki, Finland) is a new technique for IOP measurements. A very light probe is launched against the corneal surface to make a fleeting contact and spring back from the cornea. The deceleration of the probe is calculated into the IOP and displayed on the device after six rebounds per measurement [8, 9]. Due to its comfortableness and ease of use, iCare has gained much attention in clinical practice [10], especially in children [11]. Non-contact tonometer (NCT) is developed over 30 years [12]. With the advantages of not requiring topical anesthetics and fluorescein dye and no direct contact with the cornea, NCT is still a widely used screening tool for glaucoma in China and some other countries [13, 14]. Previous studies have shown that NCT measurement can provide accurate IOP assessment comparable to GAT [15]. However, these IOP measurement methods are all affected by CCT, not only for iCare and NCT but also for GAT [16].

At present, there are several tonometers available for the IOP measurement. The agreement among NCT, rebound tonometer, and GAT has been reported previously [9, 16, 17]. However, the results remain controversial due to the diversity of the study population and the difference in the IOP range of the subjects. How can we choose a more reasonable method according to the patient’s situation? Are the results of these tonometers interchangeable? What about the inter-device agreement in different IOP group? Which measurement method is more significantly affected by CCT? To the best of our knowledge, these details have not been investigated in Chinese patients.

The purpose of this study was to compare the IOP values measured by NCT, iCare pro and GAT over a wide range of IOP. With GAT as the reference tonometer, the agreement and variability with CCT were compared between NCT and iCare, to evaluate the accuracy and reliability of the three IOP measurement methods.

## Methods

### Subjects

This was a cross-sectional study, including 200 eyes of 200 subjects (76 males and 124 females). All patients were recruited from the eye center, the second affiliated hospital of Zhejiang University. The exclusion criteria were: central corneal opacities, corneal astigmatism (> 3-dimensional), nystagmus, keratoconus, ocular infection, dry eyes, pregnancy, ocular trauma, cornea with contact lenses, any history of laser refractive or intraocular surgery within the previous 3 months. The study was conducted in accordance with the Declaration of Helsinki. Ethical approval was obtained from the Ethics Committee of the second affiliated hospital of Zhejiang University. Written Informed consent was obtained from all subjects before all procedures.

### Study design

All patients underwent the following ophthalmologic examinations on the same day. Firstly, a slit lamp examination was performed on the study eyes. Secondly, IOP measurement was performed in a sitting position, using an NCT–iCare Pro–GAT sequence. All the instruments were regularly calibrated according to the manufacturers’ instructions. A 10 min break was set between each IOP measurement to minimize after-measurement fluctuations in the IOP.

NCT was first performed in each subject with an air puff tonometer (Topcon CT-80, Topcon Corporation, Tokyo, Japan). Three consecutive measurements within 2 mmHg on each eye were performed. If they differ by more than 2 mmHg, another measurement was obtained. The average of three final measurements was taken for analysis. During the iCare pro (iCare Finland Oy, Helsinki, Finland) measurement, a disposable, single-use probe was loaded into the device and aligned 4–8 mm perpendicular to the central cornea. Six consecutive measurements were performed. The software automatically discarded the highest and the lowest values, and the IOP was calculated from the remaining four values. Only proper measurements (the green background indicating within reasonable limits) were accepted. GAT (GAT AT900, Haag Streit, Koniz, Switzerland) was performed with the Goldmann applanation device mounted on a slit–lamp biomicroscope. After installation of a drop of 0.25% fluorescein with 0.5% proparacaine hydrochloride (Alcaine, Alcon, Couvreur, Belgium) in each eye, three sequential measurements were performed. If the results were within 2 mmHg, no further testing will be performed, and the final IOP will be the average value of three measurements.

Then, central corneal thickness (CCT) was measured by a solid-tip, ultrasonic pachymeter (Tomey SP-3000, Tokyo, Japan). After installation of a drop of 0.5% proparacaine hydrochloride, nine readings were performed in rapid succession and the average value was recorded. Finally, axial length (AL) was measured by IOL–Master (Carl Zeiss Meditec, Jena, Germany). IOP measurement was conducted by experienced ophthalmologists. CCT and AL was measured by skilled technicians in our ophthalmic center. All of them were independent and conducted each measurement in separate rooms. The results were cross-masked during the measurement process.

In this study, we obtained the IOP, CCT and AL values from both eyes of each patient, right eye first and then left eye. However, only the IOP value from the right eye was enrolled for analysis in order to minimize systematic bias [18]. Based on the IOP of GAT measurement, all the subjects were divided into four groups: low IOP group (eyes with IOP less than10 mm Hg); normal IOP group (eyes with IOP within the range of 10–21 mmHg); moderate elevated IOP group (eyes with IOP within the range of 22–30 mmHg); and higher IOP group (eyes with IOP over 30 mmHg).

### Statistical analysis

Descriptive statistics were performed to calculate the demographic characteristics of the study cohort. The data were expressed as mean values including the standard deviation (SD) and the 95% confidence interval (CI). Mean IOP measurements between iCare, NCT, and GAT were compared by Oneway ANOVA. Pearson correlation and intraclass correlation were used to evaluate the correlation and consistency between the three IOP measurement instruments [19]. Pearson correlation coefficient *r* = 0–0.2 indicates very week or no correlation, *r* = 0.2–0.4 indicates week correlation, *r* = 0.4–0.6 indicates moderate correlation, *r* = 0.6–0.8 indicates strong correlation, and *r* = 0.8–1.0 indicates very strong correlation. Intraclass correlation coefficient (ICC) ranges from 0 to 1. Usually, ICC < 0.2 indicates poor consistency, 0.2–0.4 indicates general consistency, 0.4–0.6 indicates moderate consistency, 0.6–0.8 indicates strong consistency, 0.8–1.0 indicates very strong consistency [20]. Bland-Altman analysis was applied to assess the agreement between the IO*P* values measured by three methods. The influence of CCT on each IOP measurement methods was evaluated by linear regression analysis. All analyses were performed using GraphPad Prism 5.0 software (GraphPad Software Inc., SanDiego, CA, USA) and SPSS for Windows, version 17.0 (SPSS Inc., Chicago, IL). A *P* value of less than 0.05 was considered to be statistically significant. Effect sizes (ESs) were also calculated in different statistical analysis to assess the magnitude of significance (Additional file 1) [21].

## Results

In this study, 200 subjects (76 males and 124 females) were recruited, with a mean age of 56.9 ± 18.3 years (range: 19–86 years). The baseline demographics of the study cohort were shown in Table 1. Among these patients, 80 eyes with an IOP > 21 mmHg, including 36 eyes with primary open angle glaucoma, 34 eyes with primary angle closure glaucoma, and 10 eyes with secondary glaucoma. The remaining 120 subjects with IOP ≤ 21 mmHg were patients with cataract (104 eyes), or normal tension glaucoma (12 eyes) or patients underwent Class surgery (CO_2_ laser-assisted sclerectomy surgery) over 3 months ago (4 eyes).

**Table 1:** Baseline demographics and mean IOP of the study population

	N	Age	NCT (mm Hg)		iCare Pro (mm Hg)		GAT (mm Hg)		CCT (μm)	AL (mm)
			Mean ± SD	range	Mean ± SD	range	Mean ± SD	range		
Total	200	56.9 ± 18.3	21.4 ± 12.0	4.0–62.4	21.0 ± 11.5	6.1–62.0	20.7 ± 11.6	4.0–63.3	537.8 ± 36.7	24.1 ± 2.1
Male	76	51.8 ± 19.2	24.9 ± 13.9	4.0–62.4	24.7 ± 14.0	6.5–62.0	24.2 ± 14.2	4.0–63.3	541.2 ± 35.6	24.8 ± 2.5
Female	124	60.1 ± 17.0	19.3 ± 10.2	6.5–56.1	18.7 ± 9.0	6.1–50.8	18.5 ± 9.1	6.0–50.6	535.8 ± 37.4	23.6 ± 1.6

*NCT* Non-contact tonometer, *iCare* rebound tonometer iCare Pro, *GAT* Goldmann tonometry, *IOP* Intraocular pressure, *SD* Standard deviation, *CCT* Central corneal thickness, *AL* Axial length

**Fig. 1:**
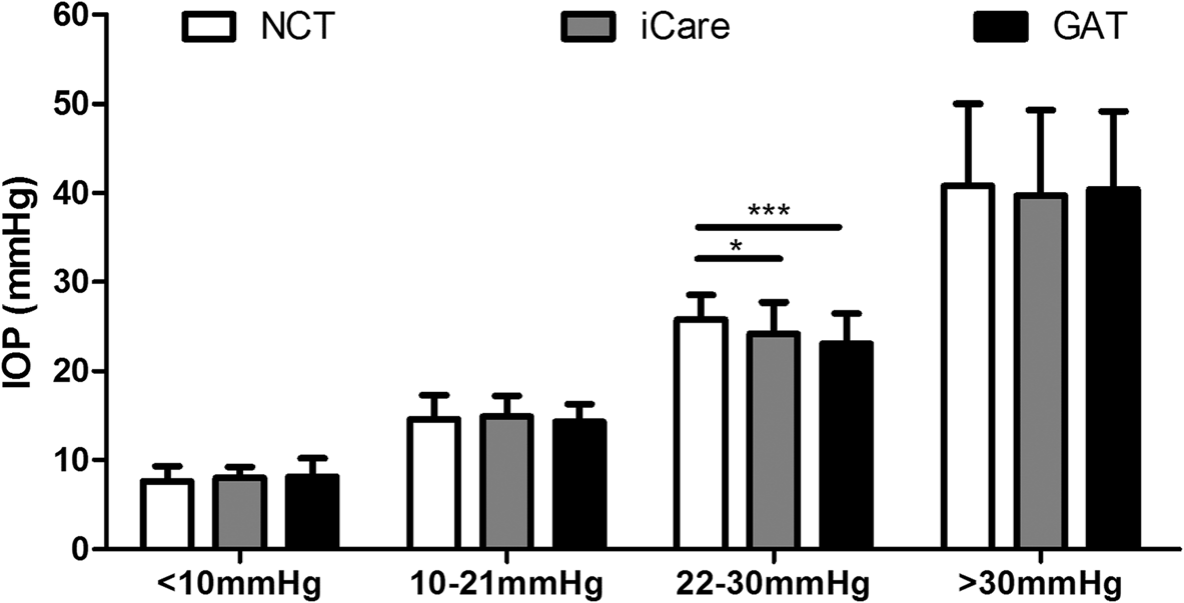
IOP measured by NCT, iCare pro and GAT in four IOP groups

The mean IOP measured by NCT, iCare Pro and GAT were 21.4 ± 12.0 mmHg (range: 4.0–62.4 mmHg), 21.0 ± 11.5 mmHg (range: 6.1–62.0 mmHg) and 20.7 ± 11.6 mmHg (range: 4.0–63.3 mmHg), respectively (Table 1). No significant difference was found between the IOP measured by three methods in IOP < 10 mmHg, IOP 10–21 mmHg, and IOP > 30 mmHg groups (*P* > 0.05, oneway ANOVA). In IOP 22–30 mmHg group, there were significant difference between the three methods (*P* = 0.001, and effect size = 0.113, 90% CI = 0.031 to 0.195). The IOP value measured by NCT was significantly higher than iCare (*P* = 0.023, and effect size = 0.533, 95% CI = 0.081 to 0.973) and GAT (*P* = 0.000, and effect size = 0.894, 95% CI = 0.432 to 1.352). However, there was no significant difference in IOP values measure by iCare and GAT (Fig. 1).

**Fig. 2:**
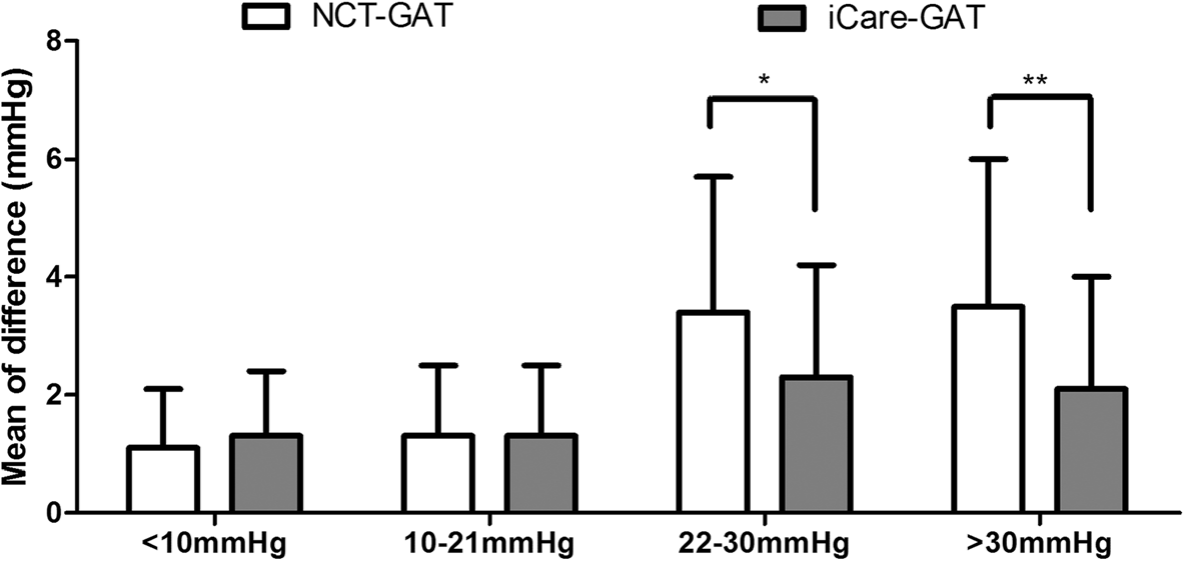
The difference of IOP measured by NCT and iCare pro compared with GAT in four IOP groups

Table 2 showed the mean value of IOP measured by the three methods in four groups. With GAT as the reference tonometer, the mean difference (Δ) of NCT–GAT and iCare–GAT in IOP < 10 mmHg group was 1.1 ± 1.0 mmHg (95% CI: 0.6–1.6) and 1.3 ± 1.0 mmHg (95% CI: 0.7–1.8). In IOP 10–21 mmHg group, the mean difference (Δ) of NCT–GAT and iCare–GAT was 1.3 ± 1.2 mmHg (95% CI: 1.1–1.5) and 1.3 ± 1.2 mmHg (95% CI: 1.1–1.5), respectively. No significant difference was found between the mean difference (Δ) in the two groups. However, in IOP 22–30 mmHg group and > 30 mmHg group, the mean difference (Δ) of NCT–GAT (3.4 ± 2.3 mmHg, 95% CI: 2.6–4.1; 3.5 ± 2.5 mmHg, 95% CI: 2.7–4.3) was significantly higher than iCare–GAT (2.3 ± 1.9 mmHg, 95% CI: 1.7–3.0; 2.1 ± 1.9 mmHg, 95% CI: 1.4–2.7, Mann Whitney test, *P* = 0.027, effect size = 0.723, 95% CI = 0.089 to 1.369 and *P* = 0.008, and effect size = 0.885, 95% CI = 0.236 to 1.535 Table 2 and Fig. 2).

**Table 2:** Measurement of IOP by NCT, iCare pro and GAT in four IOP groups

	IOP < 10 mmHg			IOP 10–21 mmHg			IOP 22–30 mmHg			IOP > 30 mmHg		
	NCT	iCare	GAT	NCT	iCare	GAT	NCT	iCare	GAT	NCT	iCare	GAT
Mean	7.6	8.0	8.1	14.6	14.9	14.3	25.8	24.2	23.1	40.8	39.7	40.4
SD	1.7	1.2	2.1	2.7	2.3	2.0	2.7	3.5	3.4	9.2	9.6	8.8
Range	4.0–10.5	6.1–11.0	4.0–10.3	9.0–21.0	9.7–20.6	10.0–19.3	20.7–32.7	15.8–30.5	16.0–30.3	28.0–62.4	28.7–62.0	30.0–63.3
Mean^△^	1.1	1.3		1.3	1.3		3.4	2.3	*	3.5	2.1	**
SD^△^	1.0	1.1		1.2	1.2		2.3	1.9		2.5	1.9	
95% CI	0.6–1.6	0.7–1.8		1.1–1.5	1.1–1.5		2.6–4.1	1.7–3.0		2.7–4.3	1.4–2.7	
[−3,+ 3]mmHg^#^	95%	95%		88%	91%		50%	80%		53%	80%	
^△^ > ±5 mmHg^##^	0%	0%		1%	0%		22.5%	15%		30%	10%	

*NCT* Non-contact tonometer, *iCare* rebound tonometer iCare Pro, *GAT* Goldmann tonometry, *IOP* Intraocular pressure, *SD* Standard deviation

*Mean*^*△*^ Mean of differences (NCT–GAT, iCare–GAT), *SD*^*△*^ Standard deviation of differences (NCT–GAT, iCare–GAT), *P < 0.05, **P < 0.01, Mann Whitney test

^#^a tolerable difference was defined as a difference within ±3 mmHg; ^##^a significant variability was defined as a difference over ±5 mmHg

**Fig. 3:**
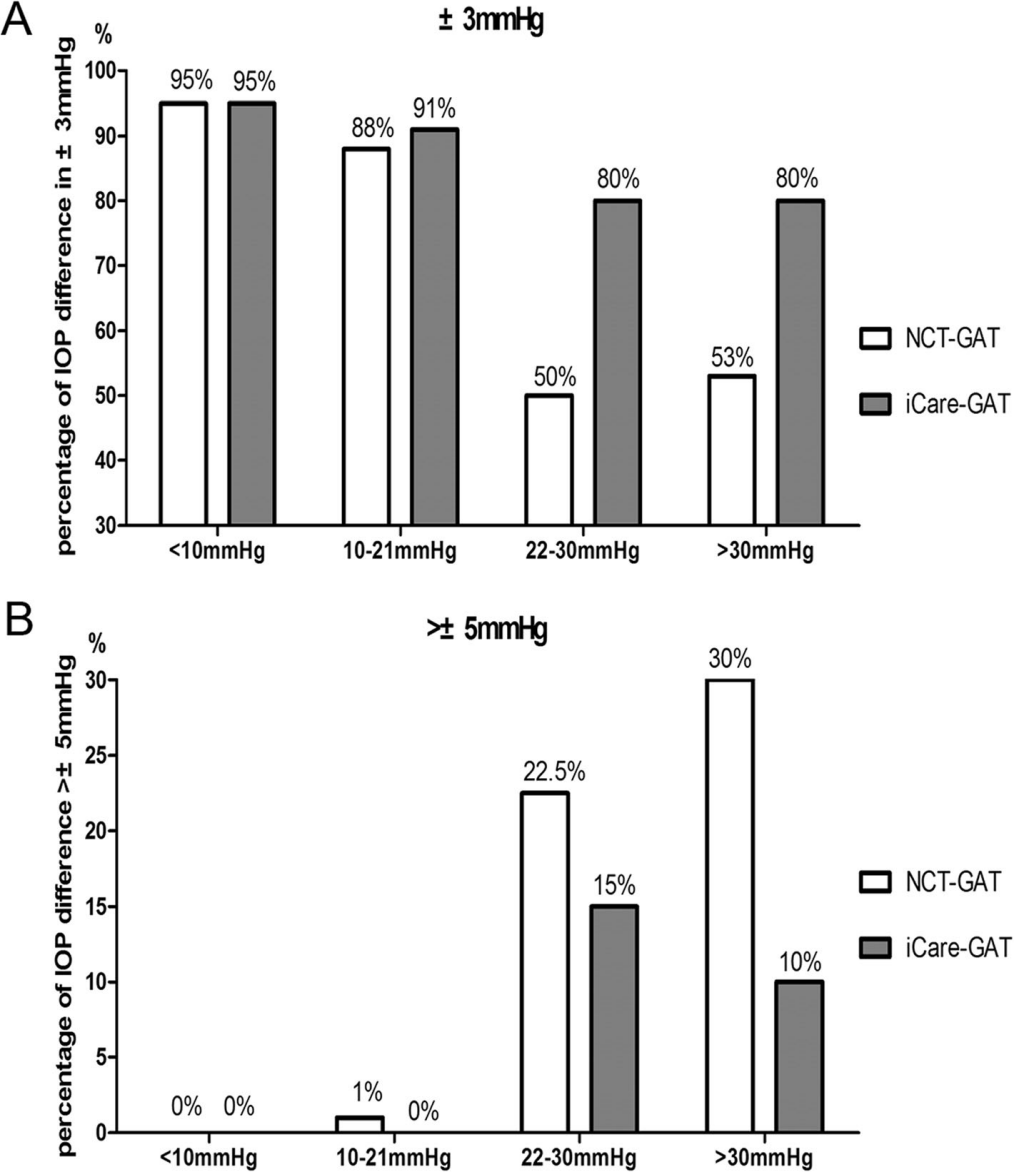
The proportional distributions of the agreements of within ±3 mmHg (**a**) and over ±5 mmHg (**b**) compared between NCT and iCare pro in four IOP groups (GAT as reference tonometer)

Compared with GAT measurements, the proportional distribution of the agreements of within ±3 mmHg measured by NCT was 95, 88, 50 and 53% in four IOP groups. By contrast, 95, 91, 80 and 80% of the readings measured by iCare pro were within ±3 mmHg (Table 2, Fig. 3a). Besides, the percentage of over ±5 mmHg deviation from GAT measurement in four IOP groups was 0, 1, 22.5 and 30% measured by NCT and 0, 0, 15 and 10% measured by iCare pro, receptively (Table 2, Fig. 3b).

**Fig. 4:**
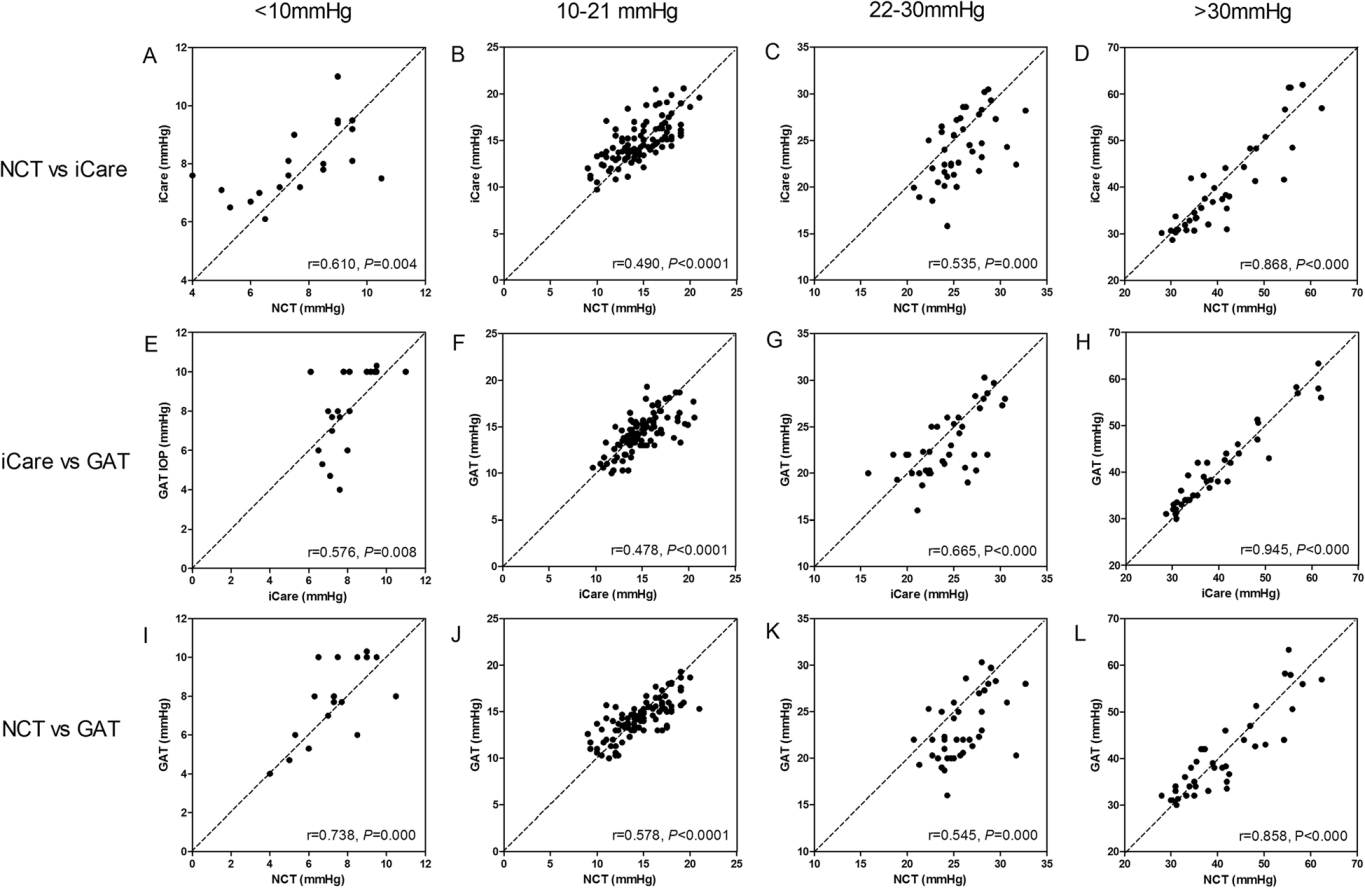
Correlation between the three IOP measurement in four IOP groups. Horizontal panel **a**-**d**: correlation between NCT and iCare pro; horizontal panel **e**-**h**: correlation between iCare pro and GAT; horizontal panel **i**-**l**: correlation between NCT and GAT; low IOP group: vertical panel **a**, **e**, and **i**; normal IOP group: vertical panel **b**, **f** and **j**; moderate elevated IOP group: vertical panel **c**, **g** and **k**; higher IOP group: vertical panel **d**, **h** and **l**

**Fig. 5:**
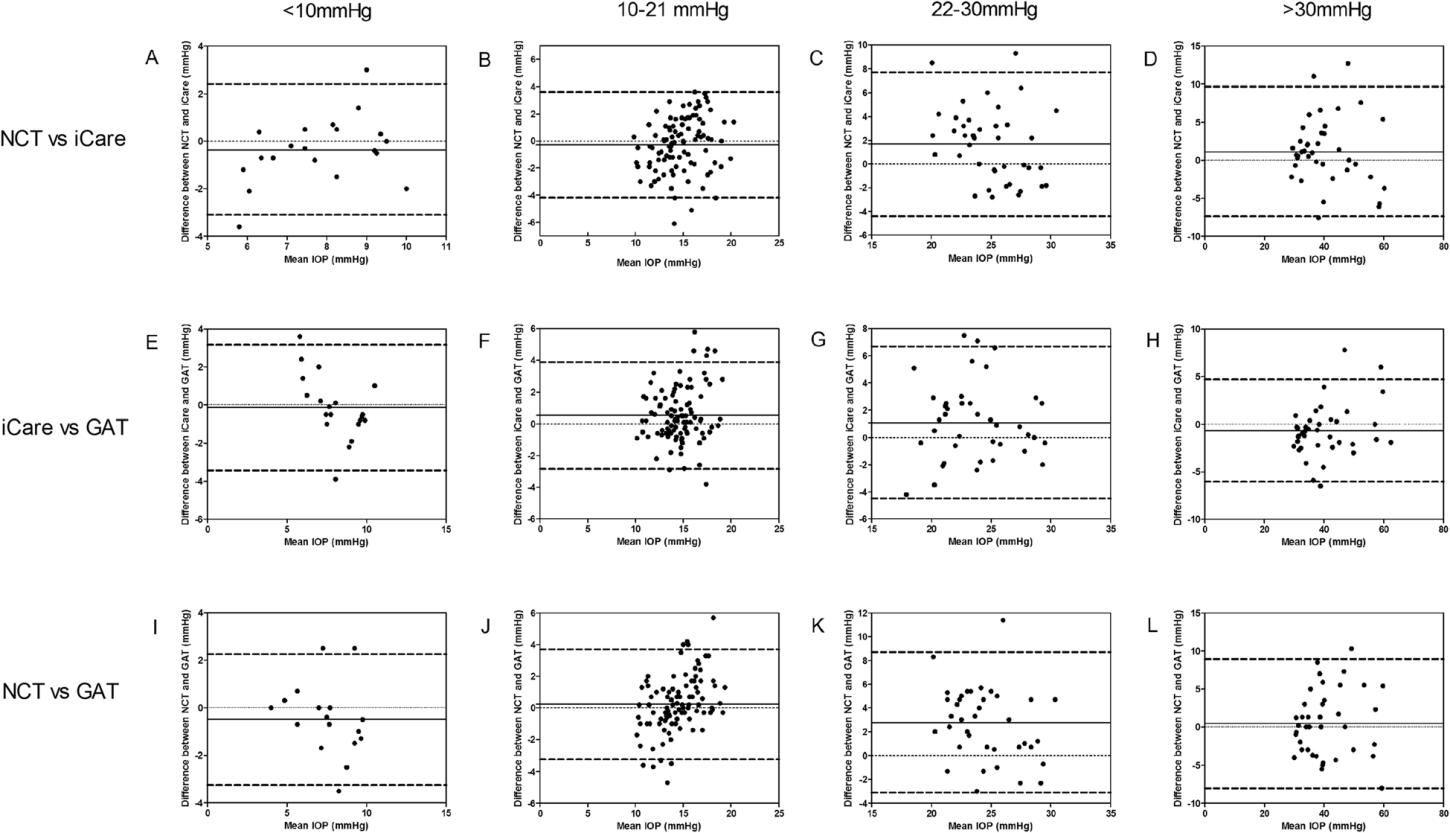
Bland–Altman plot of means against the difference between the IOP measured by NCT, iCare pro and GAT in four IOP groups. NCT vs iCare pro: horizontal panel **a**-**d**; iCare pro vs GAT: horizontal panel **e**-**h**; NCT vs GAT: horizontal panel **i**-**l**; low IOP group: vertical panel **a**, **e**, and **i**; normal IOP group: vertical panel **b**, **f** and **j**; moderate elevated IOP group: vertical panel **c**, **g** and **k**; higher IOP group: vertical panel **d**, **h** and **l**

The correlation between NCT, iCare pro and GAT measurements in four IOP groups were shown in Table 3 and Fig. 4. Pearson correlation showed a significant correlation between the three devices in each IOP group (, Fig. 4, *P* < 0.01). Intraclass correlation coefficient (ICC) also showed good consistency between the three measurement instruments (Table 3). The agreements between the three measurements were illustrated in Fig. 5 as Bland–Altman plots in four IOP groups, respectively.

**Table 3:** Pearson correlation vs intraclass correlation between NCT, iCare pro and GAT measurements in four IOP groups

		IOP < 10 mmHg			IOP 10–21 mmHg			IOP 22–30 mmHg			IOP > 30 mmHg		
		NCT	iCare	GAT	NCT	iCare	GAT	NCT	iCare	GAT	NCT	iCare	GAT
r	NCT		0.610	0.738		0.490	0.578		0.535	0.545		0.868	0.858
	iCare			0.576			0.478			0.665			0.945
ICC	NCT		0.572	0.714		0.687	0.726		0.457	0.381		0.889	0.886
	iCare			0.521			0.668			0.638			0.995

*NCT* Non-contact tonometer; *iCare* rebound tonometer iCare Pro, *GAT* Goldmann tonometry, *IOP* Intraocular pressure, *r* Pearson correlation coefficient, *ICC* Intraclass correlation coefficient

**Fig. 6:**
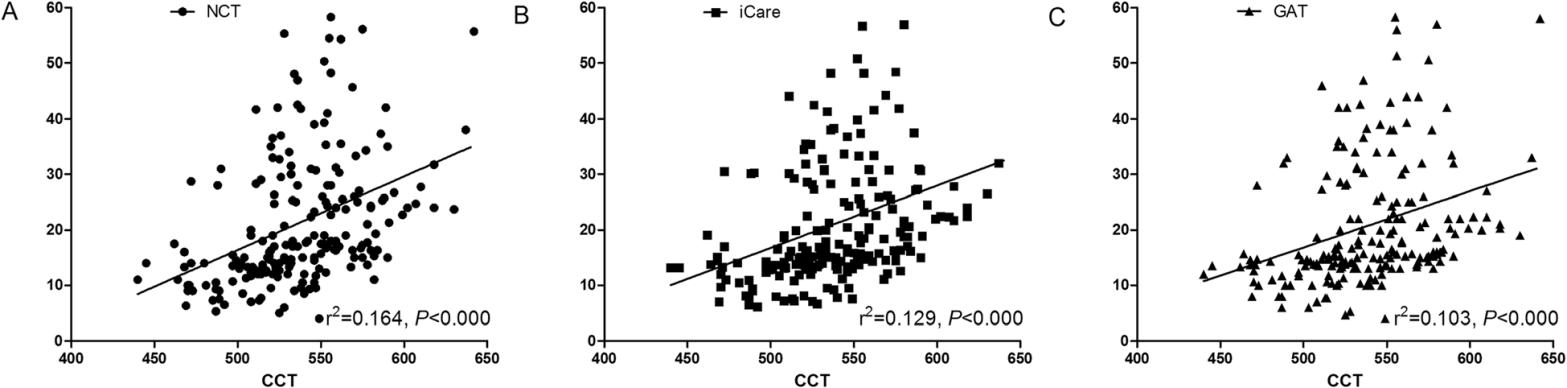
Linear regression between CCT and IOP measured by NCT (**a**), iCare pro (**b**) and GAT (**c**)

The mean CCT of the study population was 537.8 ± 36.7 μm (range: 440–642 μm). Figure 6 showed the linear regression of CCT with the IOP measured by the three devices. The IOP measurements of NCT, iCare pro, and GAT were significantly correlated with CCT. The regression coefficient were r ^2^ = 0.164 (slope: 0.132, *P* < 0.000), r ^2^ = 0.129 (slope: 0.112, *P* < 0.000) and r ^2^ = 0.103 (slope: 0.101, *P* < 0.000), respectively. A regression equation was calculated and apparent increase in IOP per 10 μm increase in CCT was 1.3 mmHg with NCT, 1.1 mmHg in iCare pro, and 1.0 mmHg in GAT over a wide range of IOP and CCT. As for the normal IOP group, an increase of IOP as 0.6 mmHg with NCT, 0.5 mmHg with iCare pro, and 0.3 mmHg with GAT for every 10 μm increase in CCT. In low and elevated IOP group, no significant correlation was found between three tonometers and CCT. Other factors such as axial length (AL), gender and age showed no significant association with IOP measured by the three methods.

## Discussion

An accurate IOP measurement is a necessary ophthalmic examination in clinical practice. In the present study, we compared the IOP measurements obtained by three methods in a wide range of IOP and confirmed significant correlations between the IOP measured by NCT, iCare pro and GAT in four groups. Bland–Altman plots also showed high agreement between the three devices in each IOP group (*P* < 0.01). These results were consistent with previous studies [16, 17].

### Advantages and disadvantages of three tonometers

GAT has always been regarded as the gold standard in the measurement of IOP [22, 23]. However, GAT has some disadvantages. First, an experienced technician and a slit lamp were needed for GAT measurement. Second, topical anesthesia and fluorescein staining were required during IOP measurement. Thirdly, the contact with the prism tip may increase the risk of infection [24]. The principle of GAT measurement is to flatten a given area of the central cornea. Therefore, GAT is inevitably affected by corneal biomechanics and central corneal thickness [4, 25]. It was reported that GAT measurement was most accurate with a CCT of 520 μm [26]. A cornea with thicker CCT results in an overestimate of IOP, while thinner CCT results in an underestimate of IOP [27].

NCT is air-puff tonometry. It does not require topical anesthetics and fluorescein dye. Since it is noncontact with the cornea, NCT can minimize the risk of cross-infection. Without the dependence of a slit-lamp biomicroscope, NCT is faster to use than GAT and can be easily performed by paramedical staff [24]. Due to these favorable characteristics, NCT is widely used not only as a screening tool in clinical practice but also in the management and follow up of glaucoma patients [28]. In China, NCT is the most popular method of IOP measurement in daily ophthalmic clinic both in general hospital and in the primary health care community. However, NCT also has some disadvantages. For example, NCT cannot be used in infants and bed-ridden patients. False readings are frequent in young children and poorly compliant patients if they squeeze their eyelids during NCT measurement. Also, there can be an error in NCT measurements in patients after penetrating corneal injury or corneal transplantation.

Fortunately, the emergence of rebound tonometer makes up for the shortcomings of GAT and NCT. This type of tonometer was first described in 1997 by Kontiola and available as iCare TA01i in 2003 [10, 29]. ICare pro is the updated version of iCare TA01i launched in 2011. It is a small, hand-held device with a single-use magnetized probe, which makes six consecutive bouncings back from the cornea during the measurement. It does not require local anesthesia and fluorescein dye. Compared with GAT and NCT, iCare pro is much easier to use for the operators and less discomfort for the patients. It can be used in both horizontal and vertical positions, which provides a unique facility for IOP measurement in sleeping children and for monitoring 24-h IOP [30, 31]. The iCare tonometer was proved to be comparable with GAT and could be considered as an alternative to GAT, especially when GAT was not available [32].

### Comparison between NCT, iCare pro and GAT

#### NCT versus GAT

In previous studies, NCT was found to be well correlated and reasonably accurate with GAT in a normal IOP range [27]. However, there was a wider bias and poor sensitivity in NCT readings compared with GAT in elevated IOP group [33]. Different NCT devices may also come out with different results. Bang et al. reported that IOP measured by Canon TX–20P and Topcon CT–1P tended to be overestimated compared with GAT, while IOP measured by Nidek NT–530P showed lower results [34]. In our study, Topcon CT–80 was applied for NCT measurement. Similar to previous studies [33], a significant difference was found in the moderate (22–30 mmHg) and higher (> 30 mmHg) elevated IOP group. The mean difference was 3.4 ± 2.3 mmHg (*P* < 0.05) and 3.5 ± 2.5 mmHg (*P* < 0.01), respectively. A “tolerable” difference of ±3 mmHg and “significant” variability of > ± 5 mmHg were defined in our study. Compared with GAT, 95 and 88% NCT readings were within ±3 mmHg in low (< 10 mmHg) and normal IOP group (10–21 mmHg). However, only half NCT readings were within ±3 mmHg in elevated IOP group. Meanwhile, the variability was significantly higher in NCT in moderate (22.5% over ±5 mmHg) and higher (30% over ±5 mmHg) elevated IOP group. These results indicated that NCT seemed to overestimate IOP in increased IOP group compared with GAT.

#### iCare pro versus GAT

iCare rebound tonometer has gained more and more interest recently. However, its accuracy of IOP measurement remains relatively controversy. Some researchers reported that IOP measured by iCare was slightly higher than GAT [35, 36]. Tamcelik et al. found that iCare pro tended to overestimate IOP in low IOP and underestimate it in high IOP compared with GAT [16]. In our study, we found no significant difference between the mean IOP measured by iCare pro and GAT in four IOP groups. Over 90% reading of iCare Pro were within ±3 mmHg in low and normal IOP range, and 80% reading were within ±3 mmHg in elevated IOP group. There was no significant variability over ±5 mmHg in low and normal IOP range, and only 10–15% readings were over ±5 mmHg in elevated IOP group. These results indicated that IOP measurement with iCare pro was highly consistent with GAT.

#### NCT versus iCare pro

So far, there were few studies compared between NCT and iCare. Most researchers focus on its agreement with GAT. Kato et al. reported that the mean IOP measured by iCare pro was significantly lower than NCT among healthy elderly subjects [9]. However, Anton et al. found good agreement between NCT and iCare pro in normal IOP range [37]. In our study, subjects with a wide range of IOP were included. Over 90% IOP results measured by iCare pro were within ±3 mmHg in low and normal IOP group, and 80% results were within ±3 mmHg in elevated IOP group, with GAT as reference tonometer. However, only 88% IOP readings measured by NCT were within ±3 mmHg, and the agreement decreased to almost 50% with the increase of IOP. The mean IOP measured by NCT was higher than iCare pro in moderately increased IOP group. With the increase of IOP, the mean difference between NCT and iCare pro significantly increased. Compared with NCT, iCare pro showed a higher agreement with GAT over a wide range of IOP in our study.

### Relationship between CCT and three tonometers

Almost all tonometers can be affected by central corneal thickness [38]. In our study, IOP measured by NCT, iCare pro and GAT were all positively correlated with CCT. NCT seemed to be influenced the most by CCT (r ^2^ = 0.164, *P* < 0.000), followed by iCare pro (r ^2^ = 0.129, *P* < 0.000) and GAT (r ^2^ = 0.103, *P* < 0.000). In previous studies, Guler et al. and Kato et al. also reported similar positive correlations between IOP measured by NCT, iCare pro and GAT with CCT [9, 39]. As estimated by a regression equation, every 10 μm increase in CCT resulted in an increase in IOP as 0.6 mmHg with NCT, 0.5 mmHg in iCare pro, and 0.3 mmHg in GAT in normal IOP group. There was a controversial disagreement about the effect of CCT on IOP measurement. Previous studies have reported 10 μm change in CCT could yield a deviation in mean IOP as 0.27 mmHg with NCT and 0.19 mmHg with GAT [24]. As for iCare pro, Brusini et al. found an increase of 0.7 mmHg for every 10 μm change in CCT [40]. But Tamcelik et al. reported only 0.097 mmHg deviation for every 10 μm change in CCT [16]. The deviation of IOP readings affected by CCT varies extremely different between different studies. Besides CCT, other biomechanical properties of the cornea such as corneal hysteresis and resistance may all contribute to the influence on the IOP measurement. Racial variation has also been reported in IOP measurement and showed a 0.18–0.7 mmHg range of correction per 10 μm change in CCT among different ethnic groups [41].

### Corneal corrected IOP in glaucoma patients

It was reported that prostaglandin analog (PGA) can decrease the CCT after a long-term topical usage, due to the degradation of collagen in the corneal stroma [42]. Totally, 96 subjects with glaucoma were included in our study. Among them, 68 (70.8%) patients underwent PGA medication for over 4 years. Detailed information about the PGA treatment was shown in Additional file 2: Table S1. IOP was corrected according to previous studies [26]. No significant difference was found between the corrected IOP measured by NCT, iCare and GAT (Additional file 2: Table S2). Maruyama et al. performed a study to investigate CCT in glaucoma patients who underwent only topical prostaglandin monotherapy for more than 4 years. Only about 10 mm of CCT decrease was observed during the 4-year follow-up period, and IOP values were unaffected. Their findings showed no correlation between the IOP reduction and CCT reduction, which indicated that CCT decrease induced by the instillation of latanoprost eye drops did not clinically affect IOP values. Therefore, IOP correction does not need to be considered in patients under PGA treatment [42].

### Limitations

There were several limitations to our study. First, the sample size of subjects included in the low and high IOP group was relatively small for subgroup analysis. Secondly, corneal biomechanics parameters were not evaluated in our study, which may interact with IOP measurement. Thirdly, subjects with corneal astigmatism (> 3-dimensional) were excluded from our study. Larger sample size with or without astigmatism needed to be included and corneal biomechanics should also be evaluated in our future studies.

## Conclusion

In conclusion, iCare pro showed a higher agreement with GAT over a wide range of IOP compared with NCT. The consistency between the three tonometers was similar in a low and normal IOP range. However, NCT shows a greater overestimate of IOP in moderate and higher IOP group. The variability of IOP measurement affected by CCT over a wide range of CCT is NCT > iCare pro > GAT. ICare pro could be used as an effective alternative to GAT, especially when GAT was not available.

## Supplementary information

**Additional file 1.** Measurement of effect size.

**Additional file 2: ** Corneal corrected IOP in glaucoma patients under prostaglandin analog (PGA) treatment. **Table S1**. Detailed information about the PGA treatment in glaucoma subjects. **Table S2**. CCT Corrected IOP in patients under PGA treatment.

## Data Availability

All data generated or analyzed during this study are included in this published article and its supplementary information files.

## Abbreviations

AL: Axial length
CCT: Central corneal thickness
CI: Confidence interval
GAT: Goldmann applanation tonometer
iCare: iCare pro rebound tonometer
ICC: Intraclass correlation coefficient
IOP: Intraocular pressure
NCT: Non-contact tonometer
SD: Standard deviation
