# Supplementary material for: Comparability of three intraocular pressure measurement: iCare pro rebound, non-contact and Goldmann applanation tonometry in different IOP group
Source: BMC Ophthalmol. 2019 Nov 14;19:225. doi: 10.1186/s12886-019-1236-5 (PMC6857285; doi:10.1186/s12886-019-1236-5)
Supplement: Supplementary file 1 — Additional file 1. Measurement of effect size. [file 12886_2019_1236_MOESM1_ESM.docx]

Additional file 1.

**Measurement of effect size**

A measure of effect size provides a standardized measure of the strength or magnitude of an effect. A statistical significance test tells us how confident we can be that there is an effect. A measure of effect size, gives us a standardized way of assessing the magnitude of the effect.

In practice, effect size is only ever likely to be calculated if the effect is already known to be statistically significant. Otherwise, there's no point in calculating the size of an effect, if there is no good reason to suppose there is any effect.

About 50 to 100 different measurements of effect size are known.

For Pearson’s correlation, Pearson’s r is widely used as an effect size. P value and r were already shown in Figure 4

For liner regression, R^2^ or Cohen’s f^2^ are commonly used as an effect size. R^2^ and P value were already shown in Figure 6.


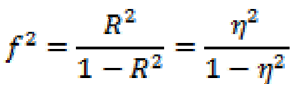


For mean difference between Groups, Cohen’s d is often used as an effect size.

For ANOVA, Eta-squared or Omega-squared are often used as an effect size.

**Table 1. Interpretation for different effect sizes in this study.**

| Effect Size | Small | Medium | Large |
| --- | --- | --- | --- |
| r | 0.10 | 0.30 | 0.50 |
| R^2^ | 0.01 | 0.06 | 0.14 |
| *η*^2^ | 0.01 | 0.06 | 0.14 |
| Cohen’s d | 0.2 | 0.5 | 0.8 |

**Table 2. Effect size, confidence interval and statistical significance of correlation between NCT, iCare pro and GAT measurements in four IOP groups.**

| Group |  | ES (r) | 95% CI | P |
| --- | --- | --- | --- | --- |
| IOP<10 mmHg | NCT *vs* iCare | 0.610 | 0.223 to 0.829 | 0.004 |
|  | iCare *vs* GAT | 0.576 | 0.179 to 0.812 | 0.008 |
|  | NCT *vs* GAT | 0.738 | 0.439 to 0.890 | 0.000 |
| IOP 10-21 mmHg | NCT *vs* iCare | 0.700 | 0.584 to 0.788 | <0.0001 |
|  | iCare *vs* GAT | 0.691 | 0.572 to 0.781 | <0.0001 |
|  | NCT *vs* GAT | 0.760 | 0.663 to 0.833 | <0.0001 |
| IOP 22-30 mmHg | NCT *vs* iCare | 0.535 | 0.268 to 0.725 | 0.004 |
|  | iCare *vs* GAT | 0.665 | 0.445 to 0.890 | <0.0001 |
|  | NCT *vs* GAT | 0.545 | 0.281 to 0.732 | 0.0003 |
| IOP>30 mmHg | NCT *vs* iCare | 0.868 | 0.759 to 0.930 | <0.0001 |
|  | iCare *vs* GAT | 0.945 | 0.896 to 0.971 | <0.0001 |
|  | NCT vs GAT | 0.858 | 0.741 to 0.924 | <0.0001 |

Effect size and confidence interval was calculated by an online calculator. https://cebcp.org/practical-meta-analysis-effect-size-calculator/
