# Supplementary material for: Comparability of three intraocular pressure measurement: iCare pro rebound, non-contact and Goldmann applanation tonometry in different IOP group
Source: BMC Ophthalmol. 2019 Nov 14;19:225. doi: 10.1186/s12886-019-1236-5 (PMC6857285; doi:10.1186/s12886-019-1236-5)
Supplement: Supplementary file 2 — Additional file 2: Corneal corrected IOP in glaucoma patients under prostaglandin analog (PGA) treatment. Table S1. Detailed information about the PGA treatment in glaucoma subjects. Table S2. CCT Corrected IOP in patients under PGA treatment. [file 12886_2019_1236_MOESM2_ESM.docx]

Additional file 2.

**Table S1. Detailed information about the PGA treatment in glaucoma subjects.**

| Glaucoma | N | PGA treatment | IOP group | PAG treatment/total |
| --- | --- | --- | --- | --- |
| POAG | 36 | 30 (83.3%) | 22-30 mmHg | 12/18 |
|  |  |  | > 30 mmHg | 18/18 |
| PACG | 34 | 28 (82.3%) | 22-30 mmHg | 16/22 |
|  |  |  | > 30 mmHg | 12/12 |
| NTG | 12 | 10 (83.3%) | < 10 mmHg | 10/12 |
| Secondary glaucoma | 10 | 0 | > 30 mmHg | 0/10 |
| Post Class | 4 | 0 | <10 mmHg | 0/4 |
| Total | 96 | 68 (70.8%) |  | 68/96 |

PGA: prostaglandin analogue; IOP: intraocular pressure; POAG: primary open angle glaucoma; PACG: primary angle closure glaucoma; NTG: normal tension glaucoma; Class: CO_2_ laser-assisted sclerectomy surgery.

**Table S2. CCT Corrected IOP in patients under PGA treatment.**

| Group |  | CCT | NCT | cNCT | iCare | ciCare | GAT | cGAT | P (ANOVA) |
| --- | --- | --- | --- | --- | --- | --- | --- | --- | --- |
| < 10mm Hg | Mean | 508.4 | 8.3 | 9.1 | 7.9 | 8.8 | 8.2 | 9.1 | 0.828 |
|  | SD | 23.9 | 1.4 | 1.6 | 0.8 | 1.2 | 1.4 | 1.4 |  |
| 22-30 mmHg | Mean | 558.6 | 26.5 | 23.7 | 24.6 | 21.9 | 24.4 | 21.7 | 0.241 |
|  | SD | 36.6 | 2.9 | 4.3 | 3.6 | 5.2 | 3.1 | 5.2 |  |
| >30 mmHg | Mean | 548.0 | 36.7 | 34.7 | 35.3 | 33.3 | 36.1 | 34.1 | 0.599 |
|  | SD | 30.4 | 5.6 | 5.5 | 5.1 | 5.5 | 4.2 | 4.7 |  |

CCT: central corneal thickness; PAG: prostaglandin analogue; NCT: Non-contact tonometer; iCare: rebound tonometer iCare Pro; GAT: Goldmann tonometry; IOP: intraocular pressure; SD: standard deviation, cNCT: corrected IOP measured by NCT; ciCare: corrected IOP measured by iCare; cGAT: corrected IOP measured by GAT.
